# Pepsin in Sea Sickness

**Published:** 1883-06

**Authors:** 


Pepsin in Sea-Sickness.—In a number of cases pepsin has proved effectual for the prevention of sea-sickness in passengers who had not made a sea-voyage before. When the first symptoms appeared, pepsin, sufficient to cover the point of a knife, was taken, followed by a glass of water accidulated with five drops of hydrochloric acid. The dose was repeated several times a day, more especially before and after meals. The favorable results obtained invite to further trials.—*Phar. Zeit.*, 1882, *No.* 20, *Ind. Blatter.*

